# Home-Based Tele-Exercise in Musculoskeletal Conditions and Chronic Disease: A Literature Review

**DOI:** 10.3389/fresc.2022.811465

**Published:** 2022-02-24

**Authors:** Adam J. Amorese, Alice S. Ryan

**Affiliations:** ^1^Baltimore Veterans Affairs (VA) Medical Center, Geriatric Research, Education and Clinical Center (GRECC), VA Maryland Health Care System, Baltimore, MD, United States; ^2^VA Research Service, Baltimore GRECC, Department of Medicine, Division of Geriatrics and Palliative Medicine, University of Maryland School of Medicine, Baltimore, MD, United States

**Keywords:** tele-exercise, home-based exercise, remote exercise, telerehabilitation, rehabilitation, exercise training

## Abstract

Exercise training is an essential component in the treatment or rehabilitation of various diseases and conditions. However, barriers to exercise such as the burdens of travel or time may hinder individuals' ability to participate in such training programs. Advancements in technology have allowed for remote, home-based exercise training to be utilized as a supplement or replacement to conventional exercise training programs. Individuals in these home-based exercise programs are able to do so under varying levels of supervision from trained professionals, with some programs having direct supervision, and others having little to no supervision at all. The purpose of this review is to examine the use of home-based, tele-exercise training programs for the treatment of different disease states and conditions, and how these programs compare to conventional clinic-based exercise training programs.

## Introduction

**Figure 1:**
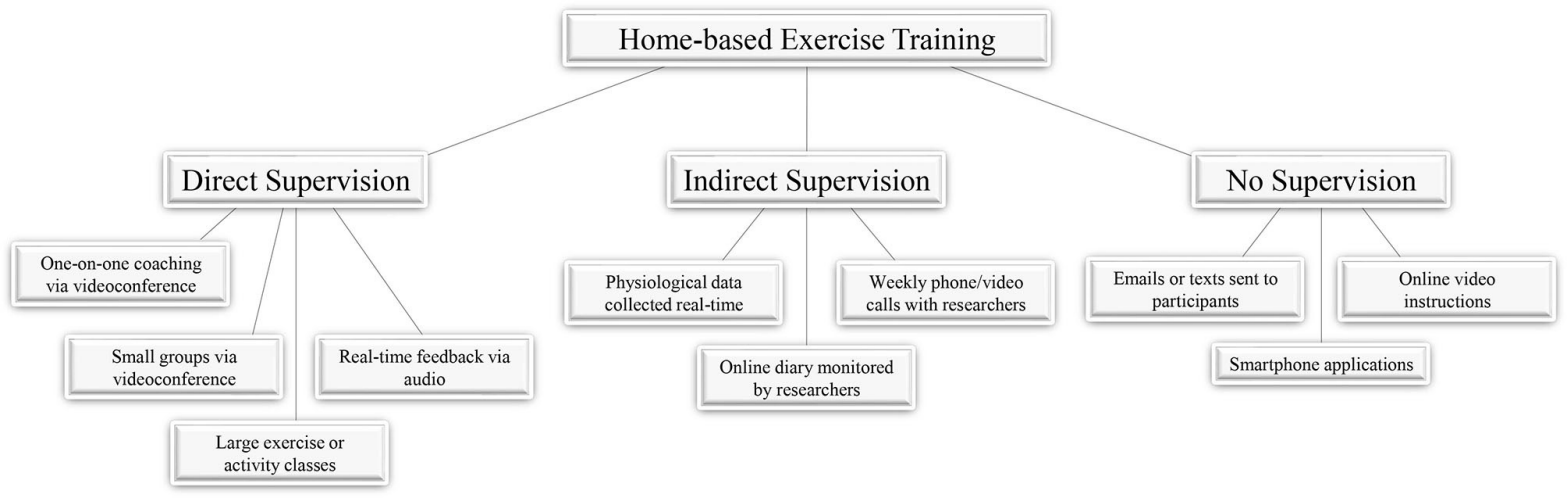
Supervision during home-based exercise. Studies employing home-based exercise as part of training or rehabilitation programs have done so with varying levels of participant supervision. The degree of supervision for exercises can range from direct supervision where participants are being monitored and communicated with in real-time, indirect supervision where participants are being contacted by researchers periodically, or no supervision where participants are given instructions or tools to engage in home-based exercises.

Although, exercise is a vital component in the treatment or prevention of numerous diseases and conditions, barriers to exercise exist in many populations (1–5). These barriers include inaccessibility to facilities, time constraints, and the cost of programs (6). One option to overcome these barriers is to implement home-based tele-exercise training to alleviate these burdens that may hinder participation in exercise programs. With advancements in technology such as high-speed internet, video-conferencing software, and smartphones, exercise training can be performed remotely under the supervision of experienced, trained professionals. Additionally, training programs may utilize methods which involve exercise with limited supervision by trained professionals, or even no supervision at all (Figure 1). These remote, tele-exercise programs may be useful in a variety of diseases or conditions, including musculoskeletal issues, diseases of the cardiorespiratory system, or neurological conditions. The following review will discuss studies employing tele-exercise training programs for the treatment and rehabilitation of these conditions and diseases.

## Literature Search

A literature search was performed from December 2020 to March 2021 on PubMed and Google Scholar with key words including but not limited to “tele-exercise,” “telerehabilitation,” and “remote exercise.” Reference lists of relevant articles were also searched. Studies included were published between 2008 and 2021 and involved home-based exercise under direct supervision during exercise, indirect supervision with some sort of contact between participants and research team members during the exercise intervention, or no supervision at all during exercise. In total, 59 studies were reviewed, with 40 of these studies being randomized control trials (RCT). Studies were organized into the following three groups: Musculoskeletal (conditions or diseases pertaining to the lower and upper extremities) (Table 1), Cardiorespiratory (conditions or diseases pertaining to cardiovascular or respiratory health) (Table 2), or Neurological (conditions or diseases pertaining to the nervous system) (Table 3).

**Table 1:** Characteristics and outcomes of home-based tele-exercise studies—musculoskeletal system.

**Research study**	**RCT/population/sample size**	**Mode of delivery/exercise**	**Intervention duration**	**Comparator groups**	**Training frequency**	**Training duration**	**Follow-up**	**Results**
Russell et al. (15)	Y/TKA/*n* = 65	Direct supervision, webcam; functional exercises	6 weeks	Telerehabilitation vs. outpatient rehabilitation	1 × /week	45 min	No follow-up	No difference in improvements in range of motion, muscle strength, pain, timed up-and-go test, quality of life, and clinical gait scores
Tousignant et al. (16)	Y/TKA/*n* = 48	Direct supervision, webcam; functional exercises	8 weeks	Telerehabilitation vs. outpatient/home visit rehabilitation	2 × /week	1 h	No follow-up	No difference in improvements in WOMAC, range of motion, balance, and lower body strength
Moffet et al. (17), Tousignant et al. (18)	Y/TKA/*n* = 205	Direct supervision, webcam; mobility, strengthening, balance, and functional exercises	8 weeks	Telerehabilitation vs. home visit rehabilitation	2 × /week	45–60 min	No follow-up	No difference in improvements in WOMAC, range of motion, and 6MWT; lower costs compared to control group
Prvu Bettger et al. (19)	Y/TKA/*n* = 306	Indirect supervision, weekly videoconference; physical therapy exercises	12 weeks	Telerehabilitation vs. outpatient/home visit rehabilitation	Unrestricted	Unrestricted	No follow-up	No significant differences between telerehabilitation and usual care in functional outcomes; lower health care costs with telerehabilitation
Bini and Mahajan (20)	Y/TKA/*n* = 29	No supervision, pre-recorded videos; physical therapy exercises	12 weeks	Telerehabilitation vs. outpatient rehabilitation	No set number of sessions	Not reported	No follow-up	No differences in improvements in clinical outcomes between groups; similar satisfaction, but lower hospital-based resource utilization in telerehabilitation group
Hinman et al. (21)	N/OA/*n* = 12	Direct supervision, webcam; strengthening exercises	3 months	Telerehabilitation only, no control group	7 total webcam sessions, 3 × /week training sessions	Not reported	3–6 months following rehabilitation	Self-reported reductions in knee pain and high satisfaction
Azma et al. (22)	Y/knee OA/*n* = 54	Indirect supervision, telephone; strengthening, endurance, flexibility, active range of motion exercises	6 weeks	Telerehabilitation vs. outpatient rehabilitation	3 × /week	Not reported	1 and 6 months	No difference in improvements in KOOS and WOMAC scores between groups at all time points
Chang et al. (23)	N/hip replacement/*n* = 31	Indirect supervision, telephone; range of motion, resistance training, walking	12 weeks	Intervention group only (pilot study)	Range of motion: 3 × /day	Range of motion: 10 min	No follow-up	Significant improvements in TUG, 6MWT, and WOMAC; high satisfaction and feasibility
					Resistance: 1 × every other day	Resistance: 10 min		
					Walking: 1 × /day	Walking: 10–30 min		
Eichler et al. (24, 25)	Y/knee or hip replacement/*n* = 111	Indirect supervision, text, telephone, and videoconference; strength and postural control exercises following inpatient rehabilitation	3 months	Telerehabilitation and usual care vs. usual care alone	3 × /week	Not reported	No follow-up	No difference in improvements between groups in functional tests such as timed up-and-go, 6MWT, and stair ascent test
Tsvyakh and Hospodarskyy (26)	N/Lower extremity injury/*n* = 74	Indirect supervision, smartphone sensors; passive and active flexion-extension, dosed-load	3 months	Telerehabilitation vs. outpatient rehabilitation	Individualized to patient	Individualized to patient	No follow-up	Higher patient satisfaction in telerehabilitation group compared to traditional rehab
Pastora-Bernal et al. (27, 28)	Y/ASD/*n* = 18	No supervision, web-based videos and images	12 weeks	Telerehabilitation vs. outpatient rehabilitation	5 × /week	Not reported	No follow-up	No difference in improvements in shoulder function between groups
Choi et al. (29)	Y/frozen shoulder/*n* = 84	No supervision, smartphone-assisted; flexion, rotation, adduction, stretching	12 weeks	Smartphone-assisted exercise vs. conventional exercise	2–3 × /day	Each exercise 10 ×	No follow-up	No difference in improvements in shoulder pain or range of motion between groups
Santello et al. (30)	Y/shoulder pain/*n* = 60	No supervision, web-based videos, instruction booklet; stretching, joint mobility, strengthening exercises	2 months	Home-based rehabilitation vs. control group receiving minimal education only	3 × /week	3–5 sets of exercises, 5–10 repetitions	No follow-up	Significant improvements in pain and disability compared to no rehabilitation
Malliaras et al. (31)	Y/rotator cuff pain/*n* = 36	Direct supervision, webcam; range of motion exercises	12 weeks	Advice only vs. recommended care vs. recommended care with telerehabilitation	1 × /week (telerehabilitation sessions)	30–60 min	No follow-up	High retention and acceptable adherence with telerehabilitation; general improvement in indices of pain and function
					3 × /week (recommended care)			
Eriksson et al. (32, 33)	N/shoulder OA/*n* = 25	Direct supervision, webcam; physical therapy exercises	8 weeks	Telerehabilitation vs. outpatient rehabilitation	1–3 × /week	30–60 min	No follow-up	Greater improvements in pain and shoulder function in telerehabilitation group
Tousignant et al. (34)	N/fracture to proximal humerus/*n* = 17	Direct supervision, webcam; stretching, pain control, range of motion, strengthening exercises	8 weeks	Telerehabilitation only, no control group	1–2 × /week	30–45 min	No follow-up	Decrease in pain and improvements in range of motion and function; high satisfaction
Steiner et al. (35)	N/chronic shoulder disease/*n* = 12	Indirect supervision, Microsoft Kinect, telephone; physiotherapeutic exercises	3 months	Telerehabilitation group only, no control group	5 × /week, 2 × /day	10–15 min	No follow-up	High useability and satisfaction; improved range of motion

**Table 2:** Characteristics and outcomes of home-based tele-exercise studies—cardiorespiratory.

**Research study**	**RCT/Population/Sample Size**	**Mode of delivery/exercise**	**Intervention duration**	**Comparator groups**	**Training frequency**	**Training duration**	**Follow-up**	**Results**
Maddison et al. (39, 40), Rawstorn et al. (41)	Y/coronary heart disease/*n* = 162	Direct supervision, audio communication, smartphone and web apps; walking at 40–65% of heart rate reserve	12 weeks	Telerehabilitation vs. outpatient rehabilitation	3 × /week	30–60 min	12 weeks	Similar increases in VO_2_ max between groups, telerehabilitation group less sedentary at 12-week follow-up; high acceptability and useability of program
Kraal et al. (42, 43)	Y/coronary heart disease/*n* = 90	Indirect supervision, telephone; aerobic exercise	12 weeks	Home-based rehabilitation vs. center-based rehabilitation	2 × /week	45–60 min	1 year	Similar significant improvements in peak VO_2_ sustained at 1-year follow-up; lower costs in home-based group
Hwang et al. (44, 45)	Y/CHF/*n* = 53	Direct monitoring, webcam; aerobic and strength exercises	12 weeks	Telerehabilitation vs. outpatient rehabilitation	2 × /week	60 min	12 weeks	No difference in non-significant 6MWD improvement between groups; lower costs for home-based group
Bravo-Escobar et al. (46)	Y/coronary artery disease/*n* = 28	No supervision, remote, ECG device; treadmill and stationary bike, resistance training	2 months	Home-based mixed surveillance rehabilitation vs. outpatient rehabilitation	3 × /week	1 h	No follow-up	Significant improvements in exercise time and capacity in both groups
Fang et al. (47)	Y/percutaneous coronary intervention/*n* = 67	Indirect supervision, telephone; walking/jogging	6 weeks	Telerehabilitation vs. usual care	3 × /week	Not reported	No follow-up	Significantly greater improvements in 6MWT and quality of life with telerehabilitation
Maddison et al. (48, 50, 51), Pfaeffli Dale et al. (49)	Y/coronary heart disease/*n* = 171	No supervision, text messages; moderate to vigorous aerobic exercise	24 weeks	Mobile health rehabilitation vs. usual care	5 × /week	30 min	No follow-up	No changes to peak VO_2_ in either group; significant increase in leisure time activity and walking in mobile health group
Frederix et al. (52, 53)	Y/coronary artery disease, CHF/*n* = 126	No supervision, email and text messages; aerobic exercise	6 months	Center-based cardiac rehabilitation plus telerehabilitation vs. center-based cardiac rehabilitation alone	3 × /week	At least 30 min	2 years	Significant improvement in peak VO_2_ in telerehabilitation group, but lost at 2-year follow-up; progressive decline in peak VO_2_ in center-based group after 6 months and 2-year follow-up
Holland et al. (60, 61)	Y/COPD/*n* = 166	Indirect supervision, telephone; walking, cycling, resistance training	8 weeks	Home-based rehabilitation vs. center-based rehabilitation	2 × /week	At least 30 min	12 months	Similar improvements in 6MWD and quality of life outcomes, lost at 12-month follow-up
Hansen et al. (62, 63), Godtfredsen et al. (64)	Y/COPD/*n* = 134	Direct supervision, webcam; endurance and resistance training	10 weeks	Telerehabilitation vs. hospital-based rehabilitation	Telerehabilitation: 3 × /week	Telerehabilitation: 35 min	12 weeks, 12 months	No difference in 6MWD improvement, only sustained at 12-week follow-up in telerehabilitation group, no difference between groups at 12 months
					Hospital-based rehabilitation: 2 × /week	Hospital-based rehabilitation: 60 min		
Chaplin et al. (65)	Y/COPD/*n* = 103	Indirect supervision, web-based program, telephone; aerobic and strength training	Up to 15 weeks	Web-based rehabilitation vs. conventional rehabilitation	2 × /week	60 min	No follow-up	Similar improvement in exercise capacity and quality of life; higher dropout rate in web-based group
Bourne et al. (66)	Y/COPD/*n* = 90	No supervision, web-based videos; strengthening exercises	6 weeks	Online rehabilitation vs. face-to-face rehabilitation	2–5 × /week	10 exercises, 30–60 s each	No follow-up	Similar non-significant improvements in 6MWT and COPD assessment test
Cameron-Tucker et al. (67)	Y/COPD/*n* = 65	Indirect supervision, telephone; walking	16–20 weeks	Telerehabilitation and outpatient rehabilitation vs. outpatient rehabilitation alone	Telerehabilitation: daily	Telerehabilitation: 30 min	No follow-up	No improvement in 6MWD in either group
					Outpatient rehabilitation: 2 × /week	Outpatient rehabilitation: 1 h		
Vasilopoulou et al. (68)	Y/COPD/*n* = 147	Indirect supervision, telephone or videoconference; walking, arm and leg exercises	12 months	Outpatient and home maintenance rehabilitation vs. outpatient and hospital maintenance rehabilitation vs. usual care	Home maintenance: 144 total sessions	Not reported	No follow-up	Both home maintenance and hospital maintenance groups maintained improvements in 6MWT and peak work rate, decreased risk of COPD exacerbations, hospitalizations
					Hospital maintenance: 2 × /week			
Tabak et al. (69)	Y/COPD/*n* = 34	No supervision, smartphone app and text message; walking	4 weeks	Telerehabilitation vs. usual care	Not reported	Not reported	No follow-up	Improvement in health status in telerehabilitation group; no change in activity levels in either group
Tsai et al. (71)	Y/COPD/*n* = 37	Direct supervision, webcam; cycling, walking, strengthening exercises	8 weeks	Telerehabilitation vs. non-exercise control group	3 × /week	Not reported	No follow-up	Significant increase in exercise capacity in telerehabilitation group
Holland et al. (72)	N/COPD/*n* = 8	Direct supervision, webcam; cycling	8 weeks	Telerehabilitation only, no control group	2 × /week	30 min	No follow-up	Significant improvements in 6MWD, dyspnea, and fatigue; high program usability and safety
Zanaboni et al. (73)	N/COPD/*n* = 10	Direct supervision, webcam; aerobic treadmill exercise, strength training	2 years	Telerehabilitation following outpatient rehabilitation, no control group	3 × /week	30 min	No follow-up	Maintained 6MWD, lung capacity, health status, and quality of life, and reduced healthcare utilization

**Table 3:** Characteristics and outcomes of home-based tele-exercise studies—neurological.

**Research study**	**RCT/population/sample size**	**Mode of delivery/exercise**	**Intervention duration**	**Comparator groups**	**Training frequency**	**Training duration**	**Follow-up**	**Results**
Chumbler et al. (82, 83)	Y/Stroke/*n* = 52	Indirect supervision, telephone and email; strength and balance exercises	3 months	Telerehabilitation vs. usual care (outpatient rehabilitation)	Not reported	Not reported	No follow-up	No significant differences in physical function measures (Late-Life Function and Disability Instrument Function and Telephone Version of Functional Independence Measure) or fall-related self-efficacy in either group
Chen et al. (84)	Y/Stroke/*n* = 54	Direct supervision, webcam; occupational and physical therapy exercises, EMG-triggered neuromuscular stimulation	12 weeks	Telerehabilitation vs. conventional rehabilitation	10 × /week	60 min	12 weeks	Significant improvements in both groups in measures of disability and daily living, balance, and muscular contraction intensity
Chen et al. (85, 86)	Y/Stroke/*n* = 52	Direct supervision, webcam; occupational and physical therapy exercises, EMG-triggered neuromuscular stimulation	12 weeks	Telerehabilitation vs. conventional rehabilitation	10 × /week	80 min	12 weeks	Significant improvements in FMA and resting-state functional connectivity in both groups, maintained at 12-week follow-up
Kairy et al. (87), Norouzi-Gheidari et al. (88)	Y/Stroke/*n* = 18	No supervision, Microsoft Kinect; occupational/physical therapy exercises, upper extremity exercises including tracing, reaching, moving, and clapping	4 weeks	Usual care and exercise gaming vs. usual care alone	Usual care: 2–3 × /week	Usual care: Not reported	No follow-up	Significant improvements in activities of daily living measures and mobility and physical domains of the Stroke Impact Scale in both groups, greater (non-significant) improvements with exercise gaming
					Exercise gaming: 2–3 × /week	Exercise gaming: 30 min		
Lloréns et al. (89)	Y/Stroke/*n* = 30	No supervision, Microsoft Kinect; stepping exercises	8 weeks	Home-based VR rehabilitation vs. clinic-based VR rehabilitation training	3 × /week	45 min	No follow-up	Similar clinically meaningful improvements in gait and balance; lower costs in home-based group
Linder et al. (90)	Y/Stroke/*n* = 99	Indirect supervision, telephone; range of motion, weight-bearing, active-assistive, activities of daily living exercises	8 weeks	Home exercise vs. home exercise and robot-assisted therapy	5 × /week	3 h	No follow-up	Significant improvements in quality of life and depression scales in both groups
Sarfo et al. (91)	N/Stroke/*n* = 20	Indirect supervision, smartphone app and telephone; mobility, strengthening, dexterity, balance, and walking exercises	12 weeks	Telerehabilitation only, no control group	5 × /week	30–60 min	No follow-up	Improvements in baseline motor deficits; high adherence and satisfaction
Szturm et al. (92)	N/Stroke/*n* = 10	Indirect supervision, video game, email and telephone; game-assisted exercises using object manipulation tasks	16 weeks	Telerehabilitation only, no control group	4 × /week	20–30 min	No follow-up	Improvements to upper extremity motor ability; high feasibility and acceptability
Paul et al. (105)	Y/MS/*n* = 30	Indirect supervision, telephone and website; aerobic, strengthening, and balance exercise	12 weeks	Telerehabilitation vs. usual care	2 × /week (minimum)	Not reported	No follow-up	No significant differences in 25 ft walk between groups or within groups; high satisfaction
Paul et al. (106)	Y/MS/*n* = 90	Indirect supervision, telephone and website; aerobic, strengthening, and balance exercise	6 months	Telerehabilitation vs. active comparator	2 × /week	Not reported	3 months	No significant differences in 2-min walk test or secondary outcomes between groups or within groups; no difference in adherence between groups, which decreased over time
Tallner et al. (107)	Y/MS/*n* = 126	Indirect supervision, website, telephone, email, strength, and aerobic training	6 months	Home-based exercise vs. waitlist control	Strength: 2 × /week	Strength: 2–3 sets per exercise	No follow-up	Significant differences between groups in muscle strength, peak expiratory flow, and sports activity; high compliance that decreased over time
					Aerobic: 1 × /week	Aerobic: 10–60 min		
Keytsman et al. (108)	N/MS/*n* = 45	Direct supervision, telephone and email; high intensity cycling	6 months	Persons with MS vs. healthy controls	3 × /week	1–3 h and 60–90 s of maximal interval training	No follow-up	Similar significant improvements in peak VO_2_, reductions in body mass
Finkelstein et al. (109)	N/MS/*n* = 12	Indirect supervision, telephone and computerized system; functional strength, stretching, and balance exercises	12 weeks	Telerehabilitation only, no control group	Customized to participant	Customized to participant	No follow-up	Significant improvements in 25 ft walk, 6MWD, and balance; high satisfaction
Fjeldstad-Pardo et al. (110)	Y/MS/*n* = 30	Direct supervision, webcam; physical therapy exercises	8 weeks	Supervised telerehabilitation vs. unsupervised home exercise vs. in-person rehabilitation	Telerehabilitation and in-person rehabilitation: 2 × /week	Not reported	No follow-up	Significant improvements in gait and balance in all groups, similar improvements between telerehabilitation and in-person rehabilitation groups
					Home exercise: 5 × /week			
van der Kolk et al. (117)	N/PD/*n* = 37	Indirect supervision, telephone, tablet app, web-based videos; cycling	6 months	Home-based exercise vs. usual care	3–5 × /week	45 min	No follow-up	Significant improvement in peak VO_2_ in home-based group, high adherence and low dropout rate
van der Kolk et al. (118)	Y/PD/*n* = 130	Indirect supervision, telephone, tablet app, web-based videos; cycling	6 months	Home-based exercise vs. active control (stretching)	3 × /week	30–45 min	No follow-up	Significant improvements in peak VO_2_ and PD symptoms in home-based group
Gandolfi et al. (119)	Y/PD/*n* = 76	Direct supervision, webcam and Nintendo Wii; exercise games, balance exercises	7 weeks	VR telerehabilitation vs. clinic-based balance training	3 × /week	50 min	1 month	Statistically greater improvements in mobility, balance for clinic group, lower cost in telerehabilitation group
Lai et al. (120)	N/PD/*n* = 20	Direct supervision, webcam; strength and aerobic exercise	8 weeks	Telecoach-assisted exercise vs. self-regulated exercise	3 × /week	20–55 min, progressively increasing by week	No follow-up	Small to moderate improvements in 6MWT in telecoach group, higher attendance and time spent exercising in telecoach group
Seidler et al. (121)	N/PD/*n* = 26	Direct supervision, webcam; tango dance	12 weeks	Telerehabilitation vs. in-person instruction	2 × /week	60 min	No follow-up	Similar significant improvements in balance and motor sign severity, comparable retention and attendance rates between groups
Lai et al. (125)	N/SCI/*n* = 4	Direct supervision, webcam; aerobic exercise (upper body ergometer)	8 weeks	Teleexercise only, no control group	3 × /week	30–45 min	No follow-up	Improvement in peak VO_2_, increased daily physical activity, high adherence
Nightingale et al. (126)	Y/SCI/*n* = 21	No supervision, accelerometer and activity diary; arm crank exercise	6 weeks	Home-based exercise vs. lifestyle maintenance control	4 × /week	45 min	No follow-up	Significant improvements in peak VO_2_, physical activity, quality of life, and fatigue in home-based exercise group
Dolbow et al. (127)	N/SCI/*n* = 17	Indirect supervision, uploaded data; FES lower extremity cycling	16 weeks	Home-based exercise only, no control group	3 × /week	40–60 min	No follow-up	High exercise adherence
Dolbow et al. (128)	N/SCI/*n* = 11	Indirect supervision, uploaded data; FES lower extremity cycling	8 weeks	Home-based exercise only, no control group	3 × /week	40–60 min	No follow-up	Significant improvements in quality-of-life measures (physical and environmental domains)
Prochazka and Kowalczewski (129), Kowalczewski et al. (130)	Y/SCI/*n* = 13	Direct supervision, webcam; upper limb strengthening, FES, computer-game assisted exercises	6 weeks	FES-assisted tele-exercise vs. conventional tele-exercise therapy	5 × /week	1 h	No follow-up	Significantly greater improvements in arm and hand function in FES-assisted group
Van Straaten et al. (131)	N/SCI/*n* = 16	Direct supervision, webcam; strengthening and stretching exercises	12 weeks	Telerehabilitation only, no control group	3 × /week	3 sets of 30 repetitions for each exercise	12 weeks	Significant decreases in shoulder pain and improvements in function and strength post-intervention, sustained at 12 weeks (strength not measured at follow-up)

## Musculoskeletal

With impairments of the joints, musculoskeletal pain and hindered mobility can occur, especially in older individuals (7, 8). These impairments can include chronic ailments, such as osteoarthritis (OA), or acute injury. Following injury and repair of the joints of the lower and upper extremities, exercise is considered a critical part of the rehabilitation process (9–12). Ultimately, the goal of these exercises is to improve strength, mobility, and balance, as well as manage pain (13, 14).

Home-based exercises under varying levels of supervision have been used in studies of rehabilitation for impairments to the lower extremities, specifically total knee arthroplasty (TKA). Three RCTs have utilized functional or strengthening exercises under direct supervision, comparing outcomes in telerehabilitation vs. outpatient or home-visit rehabilitation (15–18). No differences in improvements were observed between groups for outcomes including the Western Ontario and McMaster Universities Osteoarthritis Index (WOMAC), range of motion, muscle strength, and walking ability. One of the studies also performed a cost analysis of the intervention, which showed a lower mean cost per session in the telerehabilitation group compared to the control group, mainly due to the cost of travel for those further away from the health care center (18). One RCT with limited supervision of exercise has been performed with individuals who had TKA in which they had unrestricted use (frequency and duration) of a novel virtual physical therapy system (19). Exercises were preloaded into the system and progress was monitored remotely. Following the intervention, no significant differences were observed between those using the system and those receiving usual care in knee extension and flexion, pain, gait speed, or physical function. Heath care costs were significantly lower for those receiving virtual therapy compared to those receiving clinic-based therapy in the usual care group due to significantly fewer home health and outpatient physical therapy visits and inpatient rehospitalizations (19). One other study in individuals with TKA was done using telerehabilitation without supervision, using prerecorded videos of exercises provided to participants (20). No significant differences were observed between the telerehabilitation group and those receiving conventional rehabilitation for any clinical outcomes measured including scales of pain and physical function. Additionally, satisfaction with care was similar between groups, while utilization of hospital-based resources was 60% lower in the telerehabilitation group (20). With all of these RCTs of individuals who had TKA, a telerehabilitation group was compared to conventional outpatient rehabilitation. Independent of the level of supervision, intervention duration, or type of exercise used, results showed similar improvements in outcomes between telerehabilitation and outpatient rehabilitation groups. This suggests that using telerehabilitation exercise that follow conventional, outpatient rehabilitation methodology is a comparable option for rehabilitation treatment in individuals with TKA.

Telerehabilitation studies have also been conducted in individuals with other ailments of the lower limb, including OA, knee or hip replacement, and acute injury. For individuals with OA, exercises used in telerehabilitation studies include strengthening exercises, range of motion exercises, and flexibility exercises (21, 22). One RCT of individuals with knee OA was done using indirect supervision *via* weekly telephone calls, with exercises being done to improve strength, endurance, flexibility, and range of motion. Compared to the clinic-based group, those receiving telerehabilitation had similar significant improvements in the Knee Injury and Osteoarthritis Outcome Score and the WOMAC (22). Follow-up assessments were performed 1 and 6 months post-treatment, and no significant differences in improvement were seen between groups. Another study of individuals with knee OA was done using directly supervised exercise, assessing qualitative outcomes regarding participants' experience of the program (21). Only a telerehabilitation group was assessed during the study, with participants expressing high satisfaction with the program, finding it convenient, and appreciating not having to travel to a clinic. Participants also reported reductions in knee pain as well as improvements in physical function, allowing them to be more active (21). Two studies using telerehabilitation exercises have been done for individuals receiving knee or hip replacements, with both using indirect supervision (23–25). Both studies showed significant improvements in functional measures including the timed up-and-go and 6-min walk time (6MWT), and significant improvements in WOMAC scores. For one of the studies, these improvements were similar between the telerehabilitation group and inpatient rehabilitation control group (25), while the other study was only a pilot study in which no control group was used (23). In a telerehabilitation study of individuals with injuries to lower extremities, a novel smartphone application was created that used equipped sensors that could track movement and walking activity (26). For 3 months, participants in the telerehabilitation group performed home-based exercises with passive and active flexion-extension and dosed load on the injured limb, with the smartphone attached to the leg. Outcomes measured for this study were patient satisfaction with rehabilitation as well as time spent in consultation with their orthopedic surgeon. Compared to a control group who received traditional methods of rehabilitation such as massage, myostimulation, and pool exercises, participants in the telerehabilitation group had higher satisfaction with the intervention, as well as less time spent per visit time with their orthopedic surgeon (26). Overall, these studies utilizing home-based exercise programs for rehabilitation of the lower extremities have had varying lengths, but have generally produced significant functional improvements and high satisfaction. When long-term follow ups have been performed, improvements have persisted, granted in a limited number of studies. As improvements have been comparable to traditional exercise rehabilitation therapies, these studies show home-based telerehabilitation is a viable option for those unable or unwilling to travel for in-person services.

Home-based exercise has also been used to remedy injuries of the upper extremity, particularly for ailments pertaining to the shoulder. RCTs using home-based rehabilitation exercises have been conducted with no supervision for conditions and ailments such as arthroscopic subacromial decompression (ASD) surgery (27, 28), frozen shoulder (29), and chronic shoulder pain (30). In these studies, home-based rehabilitation resulted in significant improvements in pain and function similar to conventional rehabilitation (28, 29) or greater than education only (30). One pilot study for individuals with rotator cuff pain was designed to compare the feasibility of three different internet-based interventions including advice only, recommended care, and recommended care with telerehabilitation. As this was a feasibility study, the primary outcomes were adherence, retention, and number of adverse events. Acceptable exercise adherence was seen for the telerehabilitation group only, while incidence rates of adverse events were similar between all three groups (31). In a study of individuals who recently had shoulder replacement surgery, a directly supervised telerehabilitation program was compared to a control group receiving in-person physiotherapy. Significant improvements in pain and shoulder function ability were observed in both groups; however, the telerehabilitation group experienced significantly greater improvements compared to those of the control group (32). Interviews with the participants in the telerehabilitation group revealed a positive experience with the intervention, due to the ability to still be able to interact with the physiotherapist one-on-one while performing the exercises in their own homes (33). For individuals suffering from a fracture to the proximal humerus, a pilot study consisting of a program of stretching, pain control, active range of motion, and muscle building exercise was performed to determine its feasibility. After rehabilitation, a significant decrease in self-reported pain was observed along with a significant improvement in range of motion measurements including flexion, extension, and rotation. Additionally, upper limb function measured by the Disability of the Arm, Shoulder and Hand questionnaire showed significant, clinically relevant improvement. Participants reported high overall satisfaction with the intervention regarding the services they received (34). One more pilot study of shoulder rehabilitation has examined the feasibility of a Microsoft Kinect-based telerehabilitation system where participants performed physiotherapeutic exercises. All participants showed relative improvement in shoulder range of motion and reported high useability and satisfaction with the program (35). Similar to studies of the lower extremities, home-based exercises for rehabilitation of upper extremity ailments have shown to be effective in inducing functional improvements over various study durations. In studies that have compared telerehabilitation to conventional rehabilitation, no differences in improvements have been seen. Additionally, pilot and feasibility studies of telerehabilitation in individuals with upper extremity ailments have been generally well-received, with high satisfaction and improvements in measured functional outcomes.

## Cardiorespiratory

### Cardiovascular Disease

Following an acute cardiac event, cardiac rehabilitation (CR) provides a therapy for recovery, in which a structured, hospital-based exercise prescription is given along with patient education and risk factor modification (36). While exercise prescription can vary due to each patient's functional capacity and overall condition, programs generally contain weekly, aerobic exercise sessions that are targeted to a percentage of the patient's peak heart rate and are progressively increased in intensity, dependent on tolerance (37). Although hospital-based cardiac rehabilitation has been a long-established method of therapy, home-based cardiac rehabilitation is becoming an option for individuals with limited mobility or transportation, increasing participation and access to care (38).

Studies of home-based CR have compared its efficacy to conventional CR, using both aerobic exercise alone (39–43) or a combination of aerobic and strength training exercises (44–46). These RCTs yielded positive results, independent of the degree to which participants were supervised during exercise, with similar improvements in outcomes such as peak oxygen consumption (VO_2_) (40, 43), 6-min walk distance (6MWD) (44), and exercise time and capacity (46). Of the studies that performed follow-up assessments, no differences between the home-based CR groups or conventional CR groups were observed at 12 weeks (40, 44) or 1 year (43) following the completion of CR. Two other RCTs have compared home-based CR to usual care without a dedicated exercise component, yielding mixed results. One of the studies utilized indirectly supervised walking or jogging in the home-based CR group, which resulted in significantly greater improvements in 6MWT and quality of life measures compared to usual care (47). In the other study, participants in the home-based CR group received weekly text messages encouraging them to engage in moderate to vigorous aerobic activity, but were not directly supervised by researchers (48). While the intervention was engaging and positively received (49), participants in the intervention group and control group had no differences in exercise capacity measured by peak VO_2_ following the program. The authors believe this may be due to insufficient intensity of exercise due to participants' perception of moderate to vigorous exercise. However, those in the intervention group did have significant increases in leisure time physical activity and walking, as well as self-efficacy to be active (50, 51). In one other RCT of telerehabilitation, participants were given center-based rehabilitation focused on diet, wellbeing, behavior, and exercise training followed by a period of home-based CR, or no additional intervention. Additionally, those in the home-based CR group also received the telerehabilitation intervention during the last 6 weeks of their center-based CR. Home-based CR consisted of the use of an accelerometer and a semiautomatic coaching system which provided weekly feedback on their performance *via* email or text message (52). Significant improvements in peak VO_2_ were observed for the telerehabilitation group following the end of the 6-month program but returned to baseline values at 2-year follow-up evaluations. However, participants who only received the center-based rehabilitation program had a progressive decrease in peak VO_2_ at the end of the 6-month program and at 2-year follow up, with a significantly lower peak VO_2_ at 2-year follow up compared to the tele-exercise group. Also, the average cost per patient was ~1,000 dollars lower in the intervention group compared to the control group over the course of the program (53). Nearly all studies in which CR was delivered with an exercise component were found to improve physical outcomes following the rehabilitation program. These improvements when using telerehabilitation methods were similar to those of conventional rehabilitation, or superior to usual care without an exercise intervention. While both short and long-term telerehabilitation programs had positive outcomes, all of the studies consisted of an aerobic exercise component, which would support its importance for effective CR.

### Chronic Obstructive Pulmonary Disease

Individuals with chronic obstructive pulmonary disease (COPD) can undergo pulmonary rehabilitation (PR) to improve lung function, which often includes an exercise training component. This exercise training has shown to be able to reduce dyspnea and fatigue, as well as improve quality of life and exercise tolerance (54). Modalities of exercise that have been shown to be beneficial to those with COPD include aerobic exercise training (55, 56), resistance training (57), interval training (58), and inspiratory muscle training (59). While these exercise programs are typically performed in a clinical setting, studies using exercise training programs designed for use at home or remotely have also been employed.

Multiple RCTs have been conducted in individuals with COPD, comparing telerehabilitation vs. conventional PR methods. These studies have used differing exercise modalities, including aerobic exercise (60, 61), aerobic exercise and strength training (62–65), and strength training alone (66). No significant difference in improvements were observed between groups for outcomes including 6MWD (61, 63, 66), exercise capacity (65), and quality of life (65). However, in the studies that performed long-term follow-ups, these improvements were lost in both groups following 1 year (61, 64). Two RCTs have also been conducted comparing telerehabilitation plus conventional PR vs. conventional PR alone. In one of the RCTs, participants in the telerehabilitation group established a home-based walking plan, with weekly telephone calls with nurse mentors, while those in the control group were not instructed or contacted by researchers. Following this 8-week period, both groups then underwent conventional, center-based PR. Neither group had significant improvements in 6MWD or secondary outcomes following either the initial 8 week period, or 8–12 weeks of conventional PR (67). In the other RCT using a combination of conventional PR and telerehabilitation, the effectiveness of an indirectly supervised, home-based telerehabilitation maintenance program was compared to a similar hospital-based PR maintenance program following conventional PR. Additionally, both groups were compared to a usual care group that did not receive PR and did not have a PR maintenance program. Both the home-based maintenance group and hospital-based maintenance group showed improvements in 6MWT and peak work rate following their initial PR, and these improvements were maintained following their respective maintenance programs. Also, both groups had decreased risk of COPD exacerbations and hospitalizations compared to the usual care group (68). While the effectiveness of improving outcomes was mixed in these studies of telerehabilitation of COPD patients, improvements or lack thereof were at least similar to those receiving conventional PR. Thus, when designing telerehabilitation programs for individuals with COPD, it is critical to use exercise methods that are well-established to be sure that any potential negative or null outcome is not due to insufficient exercise intensity.

Home-based PR in COPD patients has also been compared to usual care alone, with two RCTs showing generally positive results. Aerobic exercise was used in one of the RCTs, with an unsupervised home-based walking program compared to usual care alone (69). While activity levels did not significantly differ between groups, health status (including clinical status of the airways, activity limitation, emotional dysfunction) measured by the Clinical COPD Questionnaire (70) did improve in the intervention group (69). Another RCT of COPD patients comparing home-based PR vs. usual care utilized a program of both directly supervised aerobic and strengthening exercises. Following the intervention, those receiving home-based PR had significant increases in exercise capacity and self-efficacy compared to the non-exercising control group (71). While multiple studies have been conducted comparing telerehabilitation vs. conventional PR in COPD patients, two studies of telerehabilitation have been done without comparison to a control group. In a small feasibility study of home-based PR, participants with COPD underwent a cycling program supervised by a physiotherapist. Results showed high useability and safety of the program along with significant improvements in 6MWD, dyspnea, and fatigue (72). In a pilot study of COPD patients who had completed conventional PR, participants took part in a 2-year telerehabilitation maintenance program consisting of both directly supervised aerobic and strength training exercise. Following the program, participants maintained baseline levels in outcomes such as 6MWD, lung capacity, health status, and quality of life (73). In general, these studies of telerehabilitation in individuals with COPD that did not make comparisons to conventional PR showed improvements in measured outcomes from baseline. These findings, along with the findings of studies comparing telerehabilitation to conventional PR, show that telerehabilitation is a viable option for PR in individuals with COPD.

## Neurological

### Stroke

Following stroke, exercise training is recommended to improve physical health as well as risk management for secondary prevention of stroke (74). With exercise, stroke patients have shown increases in both cardiovascular fitness (75) and muscle strength (76, 77). Additionally, exercise may improve walking ability (78), quality of life (79), and fatigue (80) in these individuals. Although there are many potential benefits to exercise following stroke, long-term adherence to a training program has been relatively poor, with up to 50% of individuals stopping their program within the 1st year (81). More recently, studies of home-based exercise programs have been examined in stroke patients, which may offer an alternative, acceptable method of training.

Multiple RCTs have been conducted in stroke patients using varying types of home-based exercise interventions for rehabilitation purposes. In a RCT of adults who had previously had an ischemic or hemorrhagic stroke in the past 2 years, participants took part in a telerehabilitation program using indirectly supervised strength and balance exercises (82, 83). No significant differences were observed between the telerehabilitation group and the usual care group for primary outcome measures of physical function [motor subscale of the Telephone Version of the Functional Independence Measure (FONEFIM) and the Overall Function Component of the Late-Life Function and Disability Instrument (LLFDI)] or fall-related self-efficacy (82, 83). However, there was a significant treatment effect in the telerehabilitation group for the LLFDI Disability Component, a secondary outcome measure representing clinically meaningful improvement on participants' ability to perform life tasks (82). Another RCT of stroke rehabilitation has used a combination of occupational and physical therapy exercises along with EMG-triggered neuromuscular stimulation under direct supervision. Compared to individuals who received the same rehabilitation procedures in an outpatient setting, both groups had similar improvements in measures of disability and daily living, balance, and muscular contraction (84). A later RCT by the same research group using the same patient population and intervention assessed motor function and functional connectivity of the motor cortex areas of the brain. Following the intervention, both the telerehabilitation group and conventional rehabilitation group exhibited significant improvements in measures of motor function and resting-state functional connectivity between the bilateral primary motor cortex areas (85, 86). Two RCTs of stroke patients have used virtual reality (VR) platforms, specifically Microsoft Kinect, as a form of telerehabilitation. In one of the studies, a VR exercise gaming program without supervision along with conventional therapy was compared to conventional therapy alone (87, 88). Both interventions yielded significant improvements in activities of daily living measures and both mobility and physical domains of the Stroke Impact Scale, with greater (non-significant) improvements in the exercise gaming group (88). Stepping exercise was used as part of another VR rehabilitation program with Microsoft Kinect, with stroke patients using the system at home without supervision or in the clinic. While both groups had similar, meaningful improvements in gait and balance, those in the home-based group had lower costs of therapy (89). One more RCT in stroke patients using indirectly supervised home-exercise had participants receive either home-exercise along with robot-assisted therapy or home-exercise alone. Both groups exhibited similar significant improvements of quality of life and depression scales (90).

In addition to the RCTs using telerehabilitation that have been conducted in stroke patients, smaller pilot studies have been performed without the use of a control group. A combination of strength, balance, and walking exercise was used in a pilot study where telerehabilitation was delivered *via* a smartphone application. Following the intervention, participants had improvements in baseline motor deficits, along with high adherence and satisfaction with the program (91). One other pilot study of telerehabilitation in stroke patients utilized an indirectly supervised, novel game-assisted exercise program focused on object manipulation tasks. Participants in the study displayed improvements to upper extremity mobility, and the program was found to have both high feasibility and acceptability (92). Although varying exercise modalities have been employed in studies of stroke patients undergoing home-base exercise, these training programs have been relatively successful in improvements in functional deficits. However, as long-term follow ups were not performed, it is unknown if these functional improvements can be sustained after the completion of the prescribed exercise programs. Compliance to exercise prescription after clinic-based stroke rehabilitation has been inconsistent and less than ideal (93, 94). Thus, it is necessary that future studies in stroke patients utilizing tele-exercise programs also consider long-term follow-up and behavioral factors that will improve adherence to exercise rehabilitation.

### Multiple Sclerosis

A large number of studies examining the effects of exercise training on individuals with multiple sclerosis (MS) have been discussed in other systematic reviews (95, 96). MS is a disease of the central nervous system where progressive neurodegeneration results in neurological disability, impaired mobility, and compromised quality of life (97). Exercise training can be an effective tool to manage the functional deficits that occur with MS, helping to manage symptoms of the disease (98, 99) as well as improve walking ability (100, 101), balance (102), and fatigue (103, 104).

Studies that have examined tele-exercise interventions in individuals with MS have used a variety of methods, including aerobic training (105–108), strength training (105–107, 109), balance exercises (105, 106), and physical therapy exercises (110). A pilot study of home-based telerehabilitation in individuals with MS used an indirectly supervised program of strength, stretching, and balance exercises customized to each participant, and found significant improvements in outcomes such as 25 ft walk time, 6MWD, and balance. However, as this was a pilot study, comparisons were not made to a control group (109). A similar pilot study of telerehabilitation in individuals with MS used an indirectly supervised program of strength and balance exercises along with aerobic exercise. In this study, neither the telerehabilitation group nor a usual care group displayed changes to 25 ft walk time, but high satisfaction was reported with the telerehabilitation intervention (105). The authors followed this pilot study with a larger RCT using the same methods, but again found no significant differences between groups in primary walking and balance outcomes or secondary outcomes (106). Adherence was high among the telerehabilitation group, but decreased over time (106). A RCT in individuals with MS that focused on strength training with some aerobic training compared a home-based exercise program to a waitlist control group. Significant differences between groups was found for muscle strength, as well as for measures of lung function and physical activity (107). One directly monitored study in individuals with MS utilized a home-based, high intensity cycling program with the intention of improving fitness, exercise capacity, and body composition. Participants with MS and healthy controls had similar significant improvements in peak VO_2_, while significant reductions in body mass were observed only in individuals with MS (108). One more RCT in individuals with MS compared a supervised telerehabilitation program to both an in-person rehabilitation program and an un-supervised home-based exercise program acting as a control group. While all groups exhibited significant improvements in gait and balance measures, improvements were greater in the supervised telerehabilitation and in-person rehabilitation groups compared to the home-exercise group (110). Most of these studies using tele-exercise interventions had positive outcomes following the programs, but did not make comparisons to clinic-based or conventional exercise programs. So, while results of these studies are promising for treating MS symptoms or improving physical function, conclusions cannot be made whether tele-exercise is inferior or comparable to more established exercise programs in this population.

### Parkinson's Disease

Parkinson's disease (PD) is a progressive neurodegenerative disease that presents with a number of functional impairments including postural instability (111), balance problems (112), and gait issues (113, 114). In addition to usual therapies including medication and surgical treatments, exercise training may be a complementary option in alleviating symptoms of the disease (115). Types of exercise training that may improve health in individuals with PD include aerobic exercise, gait training, balance training, resistance training, yoga, and dance (116).

Different modalities of tele-exercise with both indirect and direct supervision have been tested in individuals with PD with positive results. A feasibility study of indirectly supervised aerobic cycling exercise was designed for adults with mild PD where participants exercised at home with virtual reality software and real-life videos and had remote coaching. Compared to participants only receiving usual care, participants in the tele-exercise group had significant improvements in peak VO_2_ along with high adherence and low dropout rate (117). A larger follow-up study using the same exercise intervention vs. an active stretching control group yielded similar results, with significant improvements in peak VO_2_ and PD symptoms only in the tele-exercise group (118). VR training has also been used as a form of telerehabilitation in individuals with PD. In a RCT using VR exercise, participants in the telerehabilitation group used Nintendo Wii to perform various mobility and balance exercises under direct supervision, while the control group received in-clinic rehabilitation using sensory integration balance training. Significant improvements in balance, gait speed, ability to modify gait, and quality of life were observed in both groups, with improvements in mobility and dynamic balance statistically greater for the in-clinic group (119). However, the practical relevance of these differences compared to the telerehabilitation group were minimal. Satisfaction of the intervention was comparable between groups, but costs were significantly lower in the telerehabilitation group (119). In a pilot study of home-based exercise in PD patients, a program of aerobic and strength training exercise was compared between a group receiving telecoaching and a group self-regulating their exercise. While all participants in the telecoach group exhibited small to moderate improvements in walking capacity, changes to walking capacity in the self-regulated group varied, with some participants decreasing. Additionally, participants in the telecoach group had higher attendance and spent more time exercising than participants in the self-regulated group (120). One more study of tele-exercise in PD patients has used group tango dance as a form of telerehabilitation. Compared to individuals receiving in-person instruction, participants in the telerehabilitation group had similar significant improvements in balance and motor sign severity. Also, attendance rates and retention were comparable between the telerehabilitation and in-person groups (121). Although there have been a limited number of studies utilizing tele-exercise in individuals with PD, the studies conducted had high participation, with comparable improvements in physical function. As the few studies have used different exercise modalities, there are many options for future research that may be viable for managing the symptoms of PD.

### Spinal Cord Injury

Because spinal cord injury (SCI) can occur along different segments of the spine, functional impairment can vary greatly, dependent on the site of injury. Depending on the type of exercise training, exercise can promote functional recovery by helping to strengthen muscles and promote motor function recovery, as well as improve aerobic capacity (122). Various modes of exercise may be utilized by persons with SCI, including electrically stimulated cycling, electrically stimulated ambulation, arm and wheelchair ergometry, resistance training, and respiratory muscle training (123, 124). Most studies involving exercise training and rehabilitation in SCI individuals have been performed in a clinical setting, but only a small number have employed tele-exercise.

For individuals with SCI, exercises used in telerehabilitation interventions have varied, including upper extremity cycling (125, 126), functional electronic stimulation (FES) lower body cycling (127, 128), and upper extremity strengthening exercises (129–131). Many of these studies have been small pilot or feasibility studies, consisting of only a tele-exercise group. The results of these studies have been generally positive, with improvements in outcomes such as shoulder pain, function, and strength (131), peak VO_2_ (125), and quality-of-life measures (128), along with high exercise adherence (125, 127). One RCT has compared two groups of individuals with SCI both receiving directly supervised tele-exercise, with one group using conventional therapy and the other group using FES-assisted therapy. While both groups had significant improvements in arm and hand functions, improvements were statistically greater in the FES-assisted group (129, 130). One other RCT in individuals with SCI has compared the use of unsupervised home-based arm crank exercise to a lifestyle maintenance control group. Outcomes that were significantly improved in the intervention group include peak VO_2_, physical activity levels, quality of life measures, and fatigue, while no significant changes were observed in the control group (126). Individuals with SCI face many barriers to exercise, including lack of accessibility to facilities and traveling restrictions (132). While the few studies using tele-exercise programs in SCI individuals have generally resulted in positive outcomes and have been well-received, sample sizes for these studies are relatively small. Future tele-exercise studies in this population are needed with a larger number of participants to verify the clinical usability of these exercise programs. As most of the tele-exercise studies performed with SCI individuals lasted 8 weeks or less, longer studies will be needed to determine feasibility of the interventions and whether the positive effects of the interventions are long lasting.

## Conclusion

Although exercise training is a vital component in the treatment of many diseases and conditions, ensuring compliance to programs can be an arduous task for clinicians. Individuals participating in these exercise training programs may lack the means to attend in-person sessions or may not want to commit time to traveling to a clinic or gym. As such, remote, tele-exercise training is showing to be a viable, alternative option. Tele-exercise studies using both direct or indirect supervision with the use of webcams, telephone calls, videos, and smartphone applications have produced comparable improvements in outcomes compared to clinic or hospital-based, in-person control interventions. In diseases or conditions where impaired mobility may hinder participation in exercise training programs, these individuals now have the opportunity to undergo exercise training in the home environment. Tele-exercise training programs can be as effective as conventional exercise training without the burden of additional travel. When measured, adherence to tele-exercise programs has been generally high, with the interventions being well-received by participants. Studies of tele-exercise interventions have ranged in duration from 4 weeks to 2 years in length, with most taking part over the course of 6–12 weeks. These studies have been effective in inducing changes to measured outcomes, but many lack a long-term follow-up to determine if these changes persist. In the studies that did perform long-term follow-up, sustainability of the changes resulting from the interventions varied. Thus, more studies utilizing tele-exercise training need to include long-term follow-ups, as sustained adherence to exercise training programs can be difficult to achieve. Regardless, tele-exercise training offers a promising tool for improving health and well-being and is becoming more viable as advancements in technology continue to progress.

## Author Contributions

AA contributed to writing original draft preparation. AR contributed to writing review and editing. AA and AR did conceptualization. Both authors approved the submitted version and final proof of the manuscript.

## Funding

This research was supported through funds by a Senior Research Career Scientist Award (ASR) from the United States Department of Veterans Affairs (VA) Rehabilitation R&D (Rehab RD) Service, VA Merit Award 001461(ASR) RR&D, VA Medical Center Baltimore Geriatric Research, Education and Clinical Center (GRECC), and National Institutes of Health Grant P30-AG028747.

## Conflict of Interest

The authors declare that the research was conducted in the absence of any commercial or financial relationships that could be construed as a potential conflict of interest.

## Publisher's Note

All claims expressed in this article are solely those of the authors and do not necessarily represent those of their affiliated organizations, or those of the publisher, the editors and the reviewers. Any product that may be evaluated in this article, or claim that may be made by its manufacturer, is not guaranteed or endorsed by the publisher.
